# A microfluidic-based approach to investigate the inflammatory response of macrophages to pristine and drug-loaded nanostructured hydroxyapatite

**DOI:** 10.1016/j.mtbio.2022.100351

**Published:** 2022-07-07

**Authors:** Sarah-Sophia D. Carter, Abdul-Raouf Atif, Anna Diez-Escudero, Maja Grape, Maria-Pau Ginebra, Maria Tenje, Gemma Mestres

**Affiliations:** a Division of Biomedical Engineering, Department of Materials Science and Engineering, Science for Life Laboratory, Uppsala University, 751 22, Uppsala, Sweden; b Ortholab, Department of Surgical Sciences-Orthopaedics, Uppsala University, Uppsala, 751 85, Sweden; c Biomaterials, Biomechanics and Tissue Engineering Group, Departament de Ciència i Enginyeria de Materials, Universitat Politècnica de Catalunya (UPC), 08930, Barcelona, Spain; d Barcelona Research Center in Multiscale Science and Engineering, Universitat Politècnica de Catalunya, 08930, Barcelona, Spain; e Institute for Bioengineering of Catalonia (IBEC), Barcelona Institute of Science and Technology (BIST), Baldiri Reixac 10-12, 08028, Barcelona, Spain

**Keywords:** Biomaterial, Calcium phosphate cement, Drug release, In vitro, Macrophage, On-chip

## Abstract

The *in vitro* biological characterization of biomaterials is largely based on static cell cultures. However, for highly reactive biomaterials such as calcium-deficient hydroxyapatite (CDHA), this static environment has limitations. Drastic alterations in the ionic composition of the cell culture medium can negatively affect cell behavior, which can lead to misleading results or data that is difficult to interpret. This challenge could be addressed by a microfluidics-based approach (*i.e.* on-chip), which offers the opportunity to provide a continuous flow of cell culture medium and a potentially more physiologically relevant microenvironment. The aim of this work was to explore microfluidic technology for its potential to characterize CDHA, particularly in the context of inflammation. Two different CDHA substrates (chemically identical, but varying in microstructure) were integrated on-chip and subsequently evaluated. We demonstrated that the on-chip environment can avoid drastic ionic alterations and increase protein sorption, which was reflected in cell studies with RAW 264.7 macrophages. The cells grown on-chip showed a high cell viability and enhanced proliferation compared to cells maintained under static conditions. Whereas no clear differences in the secretion of tumor necrosis factor alpha (TNF-α) were found, variations in cell morphology suggested a more anti-inflammatory environment on-chip. In the second part of this study, the CDHA substrates were loaded with the drug Trolox. We showed that it is possible to characterize drug release on-chip and moreover demonstrated that Trolox affects the TNF-α secretion and morphology of RAW 264.7 ​cells. Overall, these results highlight the potential of microfluidics to evaluate (bioactive) biomaterials, both in pristine form and when drug-loaded. This is of particular interest for the latter case, as it allows the biological characterization and assessment of drug release to take place under the same dynamic *in vitro* environment.

## Graphical abstract

**Figure:**
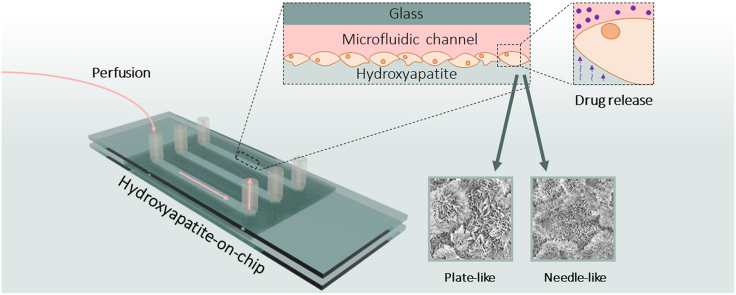

## 1 Introduction

As the global population ages, the demand for biomaterials that are able to repair, restore or replace damaged bone tissue continues to grow. Over the past few decades, calcium phosphate cements (CPCs) have attracted much attention for bone regeneration applications. CPCs are prepared by mixing a powder phase (*i.e.* one or more calcium orthophosphate powders) with a liquid phase (*i.e.* water or an aqueous solution), which results in a viscous moldable paste [1]. One of the main advantages of CPCs is their ability to set through a dissolution-precipitation reaction. This means that the material can be injected in a bone defect and afterwards set, which minimizes surgical invasion and facilitates treatment of more complex-shaped defects. The combination of their low-temperature setting and their intrinsic porosity also make CPCs promising candidates to deliver drugs or other biological compounds, with limited risk of thermal denaturation or loss of activity [[2], [3], [4]]. Loading a biomaterial with a relevant drug can be interesting for various reasons. One example is to control inflammation, which, when chronic, could cause clinical failure of the biomaterial [[5], [6], [7]].

The assessment of drug-eluting biomaterials is a challenging and multidisciplinary approach [8]. There is not only a need for thorough understanding of the properties of the biomaterial, but also of the drug and its interaction with the biomaterial when placed in a physiologically relevant environment [1,2,4]. The *in vitro* characterization of drug release is typically performed according to standards provided by the United States Pharmacopeia (USP) [9]. Generally, the drug-containing carrier is immersed in a reservoir of medium, with the dynamic nature of physiological fluids being taken into account by for example stirring, dipping or providing flow through the reservoir. Although this methodology can provide an insightful prediction of the drug release profile *in vivo,* concerns regarding the lack of physiological relevance (*e.g.* medium composition and volume) and variability in results (*e.g.* caused by geometric tolerances allowed in the set-up) have been raised, challenging the validity of these methods [10]. Moreover, it should be emphasized that the current guidelines are developed for standard pharmaceutical dosage forms and not for biomaterials, for which such standardized methods are not available [11].

Biomaterials intended for clinical application should also be evaluated for their *in vitro* biological response. This type of assessment is performed according to different guidelines, for example as set by the International Organization for Standardization (ISO). Of particular interest is ISO 10993, which provides a series of international standards for the biological characterization of medical devices. This means that the evaluation of drug release and the biological characterization of biomaterials are performed under two different testing conditions. The *in vitro* biological characterization of biomaterials is predominantly based on culturing a relevant cell type in/on a biomaterial of interest, with the biomaterial being placed in a standard tissue culture vessel, under static conditions. Alternatively, an indirect approach could be used, in which cells are exposed to biomaterial extracts. Although having provided valuable insights into the biological properties of biomaterials, it is nowadays recognized that static settings do not replicate the complexity of a fully functioning organism [12,13]. In fact, a poor correlation has been shown between the results obtained *in vivo* and results from currently used *in vitro* models for biomaterials for bone repair [14]. An example illustrating this is calcium-deficient hydroxyapatite (CDHA), which is one of the commonly encountered types of CPCs [1]. *In vivo*, CDHA has shown excellent ability to induce new bone formation [[15], [16], [17]]. However, when evaluated under conventional static *in vitro* conditions, cell viability can be compromised [[18], [19], [20], [21], [22]]. This observation has been explained by the bioactive nature of CDHA, which may lead to drastic changes in the ionic extracellular environment, thereby affecting cell behavior [[20], [21], [22], [23]]. In fact, bioactivity is not limited to ionic exchanges but also to protein interactions, which are crucial in inflammation and bone healing [24]. Importantly, the extent of ionic and protein interactions with surrounding fluids is highly governed by the microstructure and crystal morphology of CDHA, with different behaviors observed for needle-like morphologies *versus* plate-like morphologies [20,25]. Overall, neither the USP nor the ISO standards offer the necessary conditions to resemble the *in vivo* environment. Thus, optimized methods for the evaluation of biomaterials under relevant physiological conditions are highly sought.

Recently, our research group has shown the potential of microfluidic technology to characterize the biological properties of biomaterials for bone substitution/regeneration in a possibly more relevant environment [26,27]. Microfluidic technology refers to miniaturized systems that contain channels with at least one dimension at the micron-scale. Such systems allow for culturing cells on biomaterials under perfusion, thereby providing a more dynamic *in vitro* test environment compared to conventional tissue culture plastic [28]. Recently, Atif et al. integrated CDHA in a polydimethylsiloxane (PDMS)-based microfluidic system, which enabled a continuous supply of cell culture medium to the cells and biomaterial [27]. As it was demonstrated, these dynamic conditions could shield against the ionic fluctuations in the cell culture medium and overcome adverse effects on the cells. More specifically, compared to cells grown under static conditions, cells that were exposed to a continuous flow regime showed higher cell viability and superior cell proliferation.

While the USP already takes the dynamic nature of the physiological environment into account, it does not match with the typical set-up for biological characterizations (*i.e.* cell culture). Using a microfluidic approach, which is also referred to as ‘on-chip’, one would have the advantage of studying both drug release and biological properties under the same dynamic testing conditions. In addition, microfluidic systems operate at smaller scales, meaning less cell culture medium and small biomaterials can be used. Not only could this allow for a reduction in costs, but it would also address the issues around low dose therapeutics, which are tricky to assess given the large media volumes that are typically used during standard dissolution testing [29,30]. Moreover, seen from the perspective of the biological characterization, the cells are geometrically confined at a more physiologically relevant length scale, and microenvironmental parameters such as fluid shear stress, mechanical load and biochemical concentration gradients can be adjusted in a controlled manner, providing the opportunity to maintain a higher level of *in vivo* relevance [28].

The overall aim of this work was to explore the potential of microfluidics to characterize a drug-releasing biomaterial relevant for bone repair and to compare the results that are obtained from evaluating a biomaterial in a standard *in vitro* test setting, to our microfluidic approach. Thus, the inclusion of standard static cell culture on discs served to benchmark the performance of the proposed microfluidic system. Furthermore, two different CDHA substrates that are chemically identical, but varying in microstructure [31] were integrated on-chip (here referred to as CDHA-on-chip) to determine how topographically different substrates influence the inflammatory response at a cellular level, capturing at the same time the topography mediated interaction of the material with the surrounding fluids in two different experimental settings. First, we investigated the interaction of these CDHA substrates with the surrounding medium. Protein sorption of a model protein albumin and the ionic exchange of calcium and phosphate were evaluated. Next, we studied the immune response of a macrophage cell line to the substrates. The cells were characterized in terms of cell viability, cell proliferation, secretion of the pro-inflammatory cytokine tumor necrosis factor alpha (TNF-α) and cell morphology. In the second part of this study, the CDHA substrates were loaded with Trolox [32,33], a model antioxidant drug chosen in this work to further evaluate the immune response of RAW 264.7 macrophages. The release of this drug was characterized and its biological effect was assessed by evaluating the secretion of TNF-α and cell morphology.

## 2 Materials and methods

### 2.1 Preparation of CDHA substrates

#### 2.1.1 CDHA discs

A moldable CPC paste was prepared by mixing a solid phase consisting of alpha-tricalcium phosphate (α-TCP) and 2 ​wt% precipitated hydroxyapatite (Merck, ref. nr. 102143) with a liquid phase of 2.5 ​wt% disodium hydrogen phosphate (Na_2_HPO_4_; PanReac, ref. nr. 131679.1210) [34]. The two phases were mixed at a liquid to powder ratio of 0.65 ​mL/g. The starting α-TCP powder was obtained by mixing calcium hydrogen phosphate (CaHPO_4_; Sigma-Aldrich, ref. nr. C7263) and calcium carbonate (CaCO_3_; Sigma-Aldrich, ref. nr. C4830) in a 2:1 ​M ratio. Afterwards, the mixture was heated at 1400 ​°C for 15 ​h in a furnace (Hobersal, CNR-58) and subsequently quenched in air. As described in previous work, the textural properties of the resulting CDHA can be tuned by modifying the grain size of the starting α-TCP powder [34]. To obtain α-TCP powders with different particle sizes, the baked powder was milled in an agate ball mill (Pulverisette 6, Fritsch GmbB) following two milling protocols. α-TCP powder with a smaller particle size (later referred to as fine, F) was prepared by three milling steps. First, with 10 balls (ø ​= ​30 ​mm) for 40 ​min at 450 ​rpm, followed by 60 ​min at 500 ​rpm and finally with 100 balls (ø ​= ​10 ​mm) for 60 ​min at 500 ​rpm. The α-TCP powder with larger particle size (later referred to as coarse, C) was only milled once, with 10 balls (ø ​= ​30 ​mm) for 15 ​min at 450 ​rpm. Precipitated hydroxyapatite (2 ​wt %; Merck, ref. 1.02143) was added as a seed in the milled powder. The particle size distribution, analyzed by laser diffraction (Mastersizer 3000, Malvern Instruments), indicated a median size of 2.9 ​μm and 7.4 ​μm for the F and C powders, respectively (Table S.M. 1). To create CDHA discs (ø ​= ​7–8 ​mm, h ​= ​2 ​mm), the CPC pastes were shaped in custom-made 3D printed (Ultimaker 2+) polylactic acid molds and set for 4 ​h at 37 ​°C in 100% relative humidity to ensure cohesion. Afterwards, the discs were immersed in a 0.9 w/v% sodium chloride (NaCl; Sigma-Aldrich, ref. nr. S7653) solution at 37 ​°C for 10 days to allow for full transformation into biomimetic CDHA. After full transformation, the F and C powders resulted in CDHA with needle-like crystals and plate-like crystals, respectively. In subsequent sections, discs made from the F and C powders are referred to as (pristine) disc-F and disc-C, respectively. Discs with ø ​= ​8 ​mm were used in the drug release studies and discs with ø ​= ​7 ​mm in the rest of the work.

#### 2.1.2 Fabrication of CDHA-on-chip

**Fig. 1:**
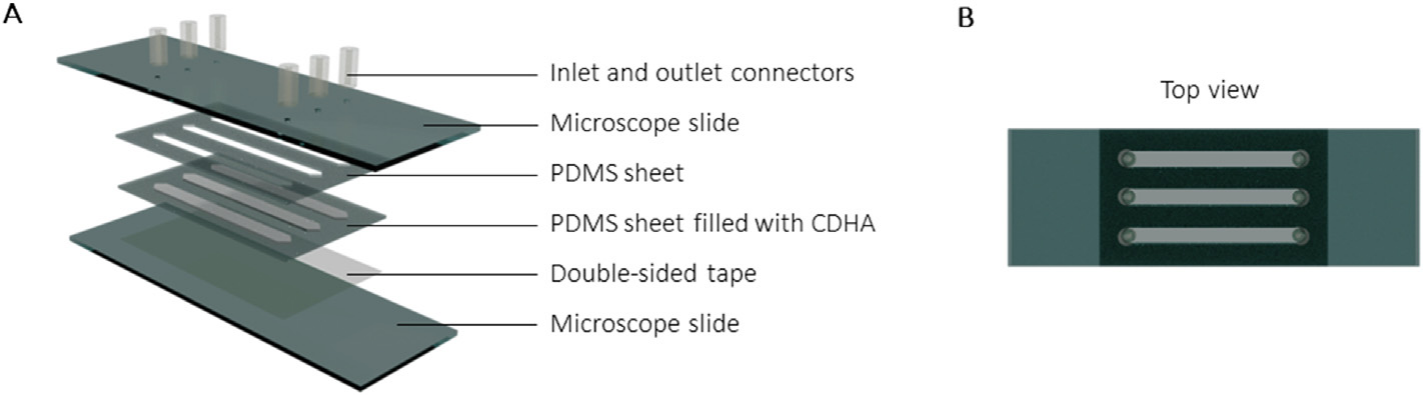
(A) Schematic of the individual layers of the CDHA-on-chip and (B) the top view of the assembled chip.

The CDHA-on-chip devices were fabricated by applying a double-sided tape (42 ​× ​25 ​mm, h ​= ​140 ​μm; Microfluidic ChipShop, Mcs-foil-008) onto a regular glass microscope slide (Fig. 1A and B). A polydimethylsiloxane (PDMS) sheet (42 ​× ​25 ​mm, h ​= ​500 ​μm; Silex Silicones), which was pre-cut to create three identical pockets using a cutter plotter (Graphtech, Craft ROBO Pro), was placed on top of this tape layer. After filling the resulting pockets (34 ​× ​3 ​mm, of which the ends are shaped in a triangle with h ​= ​1.5 ​mm, b ​= ​3 ​mm) with F or C cement paste (prepared as described in Section 2.1.1.), the chip-cement assembly was treated in the same manner as the discs. In brief, the assembly was set for up to 4 ​h and subsequently transferred to a bath containing a 0.9% NaCl aqueous solution for 10 days. After this period, an identical layer of pre-cut PDMS was plasma bonded on top of the assembly (Diener electronic GmbH, Atto Plasma Cleaner). To close the system, a microscope slide was plasma bonded on top of the second PDMS layer. The microscope glass slide contained pre-drilled holes (⌀ ​= ​1 ​mm), onto which 1 ​cm long inlet and outlet fluidic connectors (⌀_inner_ ​= ​1 ​mm, ⌀_outer_ ​= ​3 ​mm, VWR, ref. nr. 228-0701P) were glued with silicone adhesive (WACKER, ELASTOSIL® A07 Translucent). The tubing (⌀_inner_ ​= ​0.38 ​mm, ⌀_outer_ ​= ​1.09 ​mm, Portex™ Fine Bore LDPE Tubing, Smiths Medical™) was directly plugged into these connectors, linking each chip to a peristaltic pump (Shenchen, LabV1-11). The flow rate was set to 1 ​μL/min in all of the experiments. It should be noted that this flow rate was chosen to avoid excessive dilution of the medium, in order to stay within the detection limit of the ELISA kit used later in this study (Section 2.3.4.). In following sections, chips made from the F and C powders are referred to as (pristine) chip-F and chip-C, respectively.

#### 2.1.3 Preparation of Trolox-loaded CDHA substrates

Trolox-loaded CDHA substrates were prepared in a similar manner as described in Sections 2.1.1. and 2.1.2. However, 4 ​mg of Trolox (Thermo Fisher Scientific, ref. nr. 218940010) was added per 1 ​g of α-TCP powder in the solid phase of the CPC paste (0.4% g Trolox/g α-TCP). The powders were mixed with 2.5 ​wt% disodium hydrogen phosphate at a liquid to powder ratio of 0.65 ​mL/g, yielding a final Trolox concentration of 24.6 ​mM. This concentration was chosen in order to keep the concentration in the medium below 20% of the solubility of Trolox (*i.e.* sink conditions), which would avoid saturation of the cell culture medium [35]. In addition, after pre-setting, these CDHA substrates were transferred to a solution bath containing 0.9 w/v% NaCl, 6.15 ​mg/mL Trolox and 2.5 w/v% Na_2_HPO_4_. Trolox and disodium phosphate were added to avoid the release of Trolox from the samples and to maintain the pH, respectively. From now on, the Trolox-loaded substrates are referred to as disc-F-T, disc-C-T, chip-F-T and chip-C-T. It should be noted that the addition of Trolox did not have any significant effects on the micro-or nanostructure of the CDHA (Fig. S.M. 1).

### 2.2 Material analysis

#### 2.2.1 Standardization of the disc and on-chip experiments

To correlate the data obtained from the disc and on-chip samples, the surface area-to-volume-ratio (SA/V) was matched in the experiments. More precisely, the total volume passing through the chip until a certain time point was calculated, which was subsequently used to determine the amount of medium a disc sample would be exposed to. For a period of 24 ​h at the set flow rate of 1 ​μL/min, approximately 1440 ​μL of medium passed through each chip. Given that the surface area of the CDHA-on-chip was 97.5 ​mm^2^, the SA/V ratio was 0.068 ​mm^2^/μL. To match this, the discs were exposed to 1215 and 1480 ​μL of medium, for discs with ø ​= ​7 and 8 ​mm, respectively. It should be noted that the surface area of the bottom part of the discs (*i.e.* facing the bottom of the well plate) was excluded in these calculations, as it is assumed to not be in contact with the medium.

#### 2.2.2 Protein sorption by CDHA substrates

Protein sorption studies were carried out using albumin-fluorescein isothiocyanate conjugate (FITC) (Sigma-Alrich, ref. nr. A9771), which was dissolved to 25 ​μg/mL in Dulbecco's Minimum Essential Medium (DMEM) (ATCC® 30–2002™). The cell culture medium was further supplemented with 10 v/v % fetal bovine serum (FBS) (HyClone™, ref. nr. SV30160.03) and 1 v/v % penicillin/streptomycin (Gibco™, ref. nr. 15140122). Both (pristine) disc and chip samples were incubated with albumin-FITC medium for a period of 1, 6 and 24 ​h at 37 ​°C. Each disc (ø ​= ​8 ​mm) was placed in a 24-well plate and incubated with 1480 ​μL of medium. At the specified time points, the samples were washed with PBS (×3) (Gibco™ ref. nr. 14200067) and prepared for visualization. The discs were broken in half and grinded with sandpaper to obtain a flat surface. To access the CDHA on-chip, the chips were cut open at the interface between the double-sided tape and the PDMS layer. In this manner, the CDHA pieces would remain on the double-sided tape, on the bottom microscope slide. Afterwards, each channel was cut in the center to reveal its cross-section. To evaluate protein sorption, the cross-sectional area of each sample was imaged using a scanning confocal microscope (Leica, SP8; excitation ​= ​488 ​nm and emission ​= ​493–739 ​nm). The discs were placed in a well plate and the CDHA channel was held in place using a PDMS mold. The experiment was performed twice, using three samples in each condition. At least three random fields were imaged for each sample. To determine the penetration depth of albumin-FITC, the length of the stained area was measured using the measure tool in FIJI (version 1.53c). Three images were analyzed for each sample at each time point and three locations were measured in each image.

#### 2.2.3 Ionic exchange in cell culture medium

To evaluate the ionic exchange between the (pristine) CDHA substrates and the cell culture medium, the calcium and phosphate concentrations in the cell culture medium were assessed for a period of 4 days. For the disc samples (ø ​= ​7 ​mm), the medium (1215 ​μL) was collected and fully replenished every 24 ​h. On-chip, the medium was harvested from the medium collection vessel, which was fully emptied every day. The samples were kept at 37 ​°C in a humidified atmosphere with 5% CO_2_. To quantify the calcium and phosphate concentrations, colorimetric detection kits were used, following the manufacturer's instructions (Abcam, ref. nr. ab102505 and ref. nr. ab65622, respectively). In brief, every 24 ​h, the medium was collected and stored at −20 ​°C. Prior to analysis, the samples were diluted 5–10 and 100–150 times in distilled water, for the detection of calcium and phosphate, respectively. This dilution step was performed in order to remain within the limits of the standard curve. The experiment was performed twice, using three samples in each condition.

#### 2.2.4 Drug release from CDHA substrates

For the drug release studies, disc-F-T and disc-C-T samples (ø ​= ​8 ​mm, h ​= ​2 ​mm) were placed in a 24-well plate and covered in 1480 ​μL cell culture medium. After 3, 6, 9, 27, 30 and 48 ​h, a 160 ​μL aliquot was taken from each disc sample. To maintain the same initial volume, this aliquot was replaced by the same amount of fresh medium. In addition, at these same time points, all the medium was collected from the medium collection vessel of the chip-F-T and chip-C-T samples. The experiment was performed at 37 ​°C and the collected samples were stored at 2–8 ​°C until analysis. It should be noted that the samples were stored no longer than 48 ​h, and that the storage time and temperature did not affect Trolox concentration nor measurement (supplementary material, Fig. S.M. 2). Prior to the analysis, the aliquots obtained from the disc samples were diluted 1.5–6 times in cell culture medium. To determine the Trolox concentration in each of the samples, the absorbance was measured in a microplate reader at 290 ​nm (TECAN, Spark®). The obtained values were subsequently correlated to a standard curve with known concentrations of Trolox, ranging from 0 to 500 ​μM. The data was plotted in a cumulative manner and presented as the percentage of Trolox released, based on the known concentration of Trolox that was initially loaded in the substrates. For the static conditions, the dilution of the medium that was caused by the sampling process was compensated for.

### 2.3 Biological characterization

#### 2.3.1 Cell culture

The murine macrophage cell line RAW 264.7 (ATCC® TIB‒71™) was chosen as a common cell line used in immune response evaluation for biomaterials [36], and it was purchased from the American Type Culture Collection (ATCC). As per ATTC's recommendation, the cells were not passaged for more than 2 months upon thawing. As described in Section 2.2.2., the cells were maintained in DMEM, which was supplemented with FBS and penicillin/streptomycin. Upon reaching 80% confluence, the cells were dislodged from the cell culture flask using a cell scraper (Sigma-Aldrich, ref. nr. C5981) and subcultured as recommended by ATCC. The cells were kept at standard cell culture conditions of 37 ​°C in a humidified atmosphere with 5% CO_2_. The cell passage used for all experiments varied between 5 and 9 (counted the original vial received from ATTC as passage number 1).

Prior to all cell studies, the CDHA substrates without Trolox (both the discs and on-chip samples) were sterilized using 70% ethanol for 2 ​h. Subsequently, the samples were thoroughly rinsed with autoclaved distilled water (×5). To avoid the release of Trolox, the solid phase of the CPC paste used to fabricate Trolox-loaded substrates was sterilized using UV for 30 ​min. Prior to use, all samples were incubated overnight (17 ​h) with supplemented cell culture medium.

#### 2.3.2 Cell viability

To evaluate the potential of the CDHA-on-chip system to culture RAW 264.7 ​cells, cell viability was assessed for a period of 3 days. The cells were seeded at 20,000 ​cells/cm^2^, both on the discs (ø ​= ​7 ​mm) placed in 48-well plates and on the chips. As a control, the cells were seeded directly on the polystyrene surface of the well plate (PS). On the discs, the cells were seeded in 400 ​μL, matching regular cell culture conditions in 48-well plates. On-chip, the cells were seeded in a volume of 80 ​μL. After a static incubation period of 2 ​h (enough time to ensure cell attachment, Fig. S.M. 3), the discs were transferred to a 24-well plate and the medium (1215 ​μL) was fully changed. The medium was afterwards changed daily. Unidirectional perfusion was started on-chip, which continued throughout the entire experiment. After 1 and 3 days of culture, the discs were transferred to a new well and all samples were washed (×2) with transparent Minimum Essential Medium (MEM) (Gibco™, ref. nr. 51200046). The samples were subsequently stained with LIVE/DEAD staining (calcein-AM/propidium iodide (PI); Invitrogen™, ref. nr. C3099 and ref. nr. P3566, respectively) at a final concentration of 1 ​μg/mL (in transparent MEM) and incubated for 15 ​min at standard cell culture conditions while protected from light. Afterwards, the samples were washed with transparent MEM (×3) and imaged using a fluorescence microscope (Olympus, IX73; excitation/emission ​= ​494/518 ​nm and 595/615 ​nm for calcein and PI, respectively). Living cells were visualized in green and dead cells in red. The experiment was performed three times, using three samples per condition. At least three representative images were taken from each sample.

#### 2.3.3 Cell proliferation

In addition to cell viability, RAW 264.7 ​cell proliferation was evaluated using the lactate dehydrogenase (LDH) biochemical assay (Sigma-Aldrich, ref. nr. TOX7-1 ​KT). This assay can be used as an indirect method to quantify the cytosolic enzyme LDH of cells that had previously adhered to the CDHA substrate or well plate. The cells were seeded and maintained using the same procedure as described in Section 2.3.2. On day 1 and day 3, the samples were washed with PBS (×2) and subsequently lysed with 300 ​μL of 0.1 v/v % Triton-X (Sigma-Aldrich, ref. nr. T8787) for 50 ​min at 37 ​°C. This lysate was diluted twice in 0.1 v/v % Triton-X, after which a 50 ​μL aliquot was taken, transferred to a 96-well plate and incubated with 100 ​μL LDH assay reagent. After 20 ​min of incubation at room temperature while protected from light, LDH activity was determined by measuring the absorbance and background absorbance at 490 and 690 ​nm, respectively (TECAN, Spark®). The experiment was performed twice, using three samples per condition. To account for the differences in surface area between chips and discs, the obtained cell numbers were normalized to the surface area of the respective samples.

#### 2.3.4 Inflammatory response

The release of the pro-inﬂammatory cytokine TNF-α by the RAW 264.7 ​cells was evaluated using a TNF-α mouse ELISA kit (Tebu-Bio, ref. nr. ELM-TNFa). The cells were seeded on the disc, on the chips (both pristine samples and Trolox-loaded) and on the PS samples at 50,000 ​cells/cm^2^, using the same procedure as described in Section 2.3.2. This cell seeding density, which was higher than in the cell viability and proliferation studies, was chosen to avoid working below the detection limit of the ELISA kit. In addition to the above-mentioned samples, a positive control was included (PS+), by adding 1 ​μg/mL lipopolysaccharide (LPS; *Escherichia coli* O111:B4, Sigma-Aldrich, ref. nr. L-4391) to cells cultured directly on the well plate. In brief, 2 ​h after seeding (*i.e.* the static incubation time), the perfusion was started on-chip, which continued throughout the rest of the experiment. At the same time, the discs were transferred to a new well and the medium (1215 ​μL, containing LPS for the PS ​+ ​samples) was changed in all these samples. For the PS ​+ ​samples, after 2 ​h of incubation with LPS-containing medium, the PS ​+ ​samples were washed with PBS (×3) and incubated with regular supplemented medium. After 24 ​h of flow, medium aliquots were taken from all samples and stored at −20 ​°C until analysis. LPS was not added to on-chip samples to reduce the risk of bubble formation as a result of changing the medium several times. The experiment with pristine samples was performed three times, using three samples per condition. For Trolox-loaded samples, the experiment was performed twice, using three samples per condition.

For analysis, to account for the difference in cell number on the different samples, the LDH assay was performed directly after sampling for TNF-α. The samples were lysed as described in Section 2.3.3. and subsequently treated accordingly.

#### 2.3.5 Cell morphology

The cell morphology of RAW 264.7 ​cells on both the pristine and Trolox-loaded substrates was evaluated using scanning electron microscopy (SEM) (Zeiss Merlin, Germany). The cells were seeded and maintained as described in Section 2.3.2. After 1 and 3 days of culture under static conditions or flow, the samples were washed with PBS (×2) and subsequently fixed in 4% paraformaldehyde (VWR, ref. nr. 22023) for 20 ​min. The samples were rinsed in PBS (×3), dehydrated in an increasing ethanol series (10%–30% - 50%–70% - 90% - absolute ethanol) and dried overnight in a fume hood. Prior to visualization, the samples were gold-coated (2–4 ​nm) (Emitech SC7640, Quorum technologies). At least three random sites were imaged on one sample of each type.

### 2.4 Statistical analysis

The statistical analysis was performed using Minitab version 18. The data was evaluated by one-way analysis of variance (ANOVA), two-sided, at a significance level of α ​= ​0.05. Post-hoc Tukey test was performed to investigate differences between samples. When the variances were unequal (Levene's test), Welch's ANOVA was used, followed by Games-Howell test to assess differences between groups. The results are presented as mean ​± ​standard deviation from one representative experiment.

## 3 Results

### 3.1 Protein sorption

**Fig. 2:**
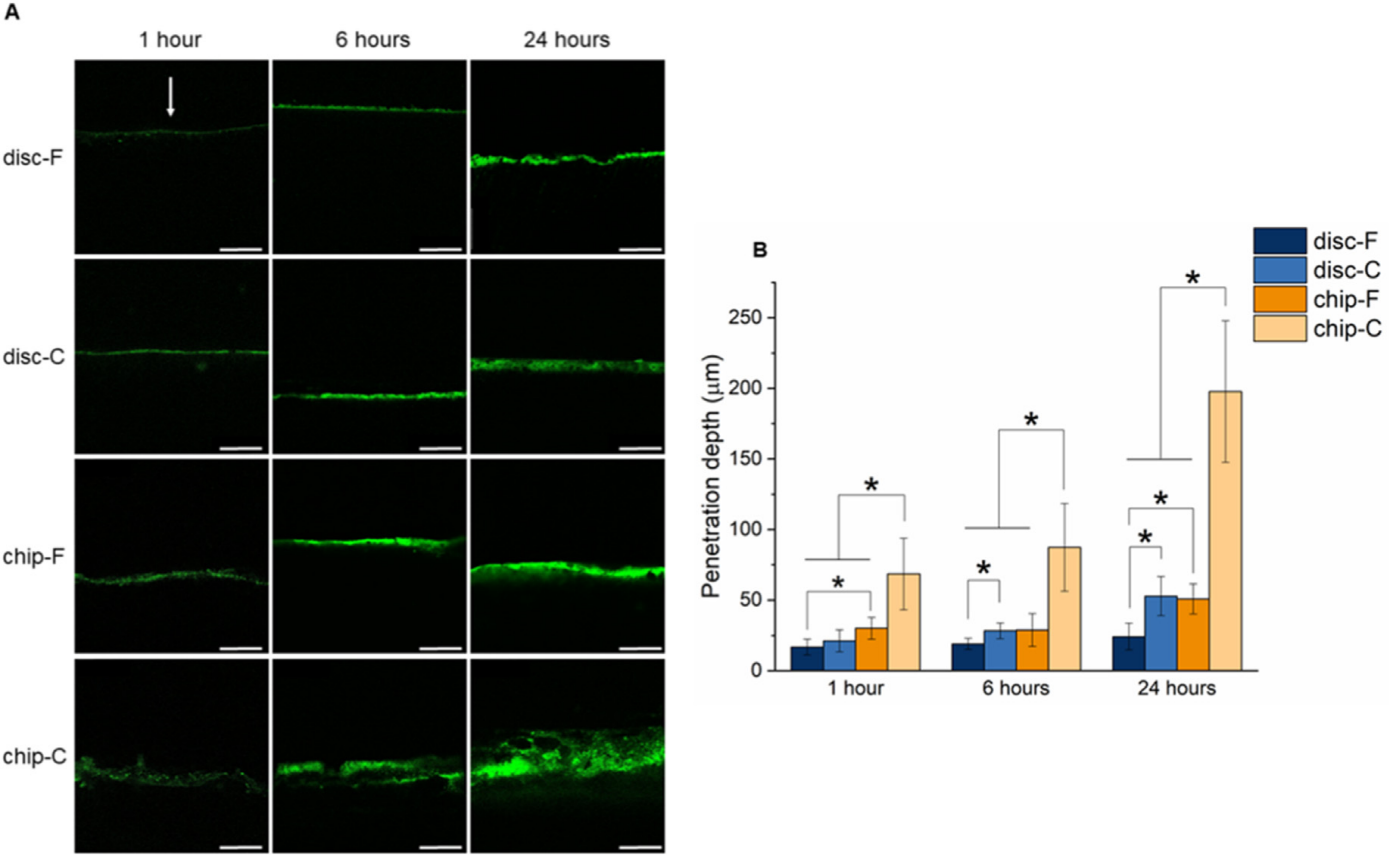
**(A)** Cross-section of disc-F, disc-C, chip-F, and chip-C samples, visualized using fluorescent confocal microscopy. Sorption of albumin-FITC solution is depicted in green. The arrow indicates the direction from which the CDHA substrates were exposed to albumin-FITC. Scale bars correspond to 250 ​μm. **(B)** Quantification of the albumin-FITC penetration depth. ∗ indicates p ​< ​0.05 between the samples.

Depending on the physico-chemical properties, CDHA materials may interact differently with the proteins in the surrounding medium. To evaluate protein sorption of the F and C substrates under static and dynamic conditions, sorption of the model protein albumin-FITC was examined. For both the discs and chips, protein sorption progressed over 24 ​h (Fig. 2A and B). In general, the sorption region was larger for the chip samples and shown to be statistically significant for the majority of the time points (p-values indicated in Table S.M. 2). Moreover, protein sorption appeared to be higher in C samples than in F samples, with statistical differences already being observed after 1 ​h for the chips and after 6 ​h for the discs.

### 3.2 Calcium and phosphate concentration in the cell culture medium

CDHA is a bioactive biomaterial, known to interact with ions that are present in cell culture medium. Since this ionic exchange can affect cell behavior [[19], [20], [21], [22], [23]], the calcium and phosphate concentrations in the medium were monitored for a period of 4 days.

**Fig. 3:**
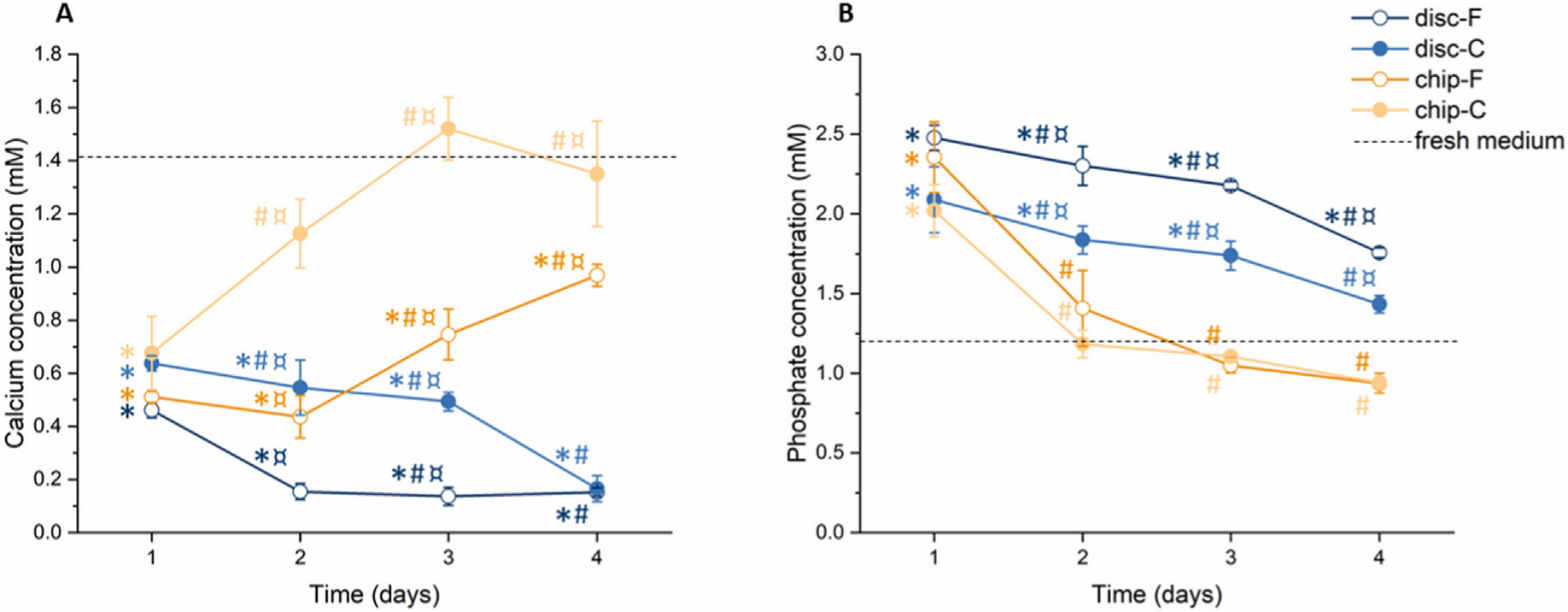
Quantiﬁcation of **(A)** calcium and **(B)** phosphate concentrations in the cell culture medium of disc-F, disc-C, chip-F and chip-C over a period of 4 days. As a control, fresh medium was included. ∗ indicates p ​< ​0.05 between the samples and fresh medium; # indicates p ​< ​0.05 between the chip and respective disc samples (*e.g.* difference between chip-F and disc-F); ¤ indicates p ​< ​0.05 between F and C substrates of the same type (*e.g.* difference between chip-F and chip-C).

As shown in Fig. 3A, after one day, both the F and C samples took up a statistically significant amount of calcium from the fresh medium, regardless the experimental set-up, *i.e.* disc or chip (p ​< ​0.0005 for all samples). However, as the experiment progressed, the calcium concentration in the medium obtained from both chip-F and chip-C continued to increase, approaching the calcium levels of fresh medium (1.41 ​± ​0.21 ​mM). For the chip-C samples in particular, from day 2 onwards, no statistically significant difference with fresh medium was observed. In contrast, for the discs, the calcium concentration in the medium continued to decrease, eventually reaching levels of 0.15 ​± ​0.02 ​mM and 1.17 ​± ​0.05 ​mM for disc-F and disc-C, respectively. From day 2, a significantly higher calcium concentration was found in chip-C samples compared to disc-C analogues (p-values indicated in Table S.M. 3). The same trend was visible for chip-F and disc-F samples starting from day 3. Generally, disc-F and chip-F depleted higher amounts of calcium from the media compared to coarse analogues (*i.e.* lower levels of calcium in the medium). More specifically, the chip-F samples took up a significantly higher amount of calcium from the media than the chip-C samples throughout the entire experimental period. A similar trend was seen for the disc-F and disc-C samples during the first 3 days, which was significant after 1 day.

Regarding the phosphate concentration, on day 1, a statistically significant amount of phosphate was released into the medium, both for the discs and chips (Fig. 3B, p-values indicated in Table S.M. 4). Overall, with time, the phosphate concentration decreased towards the levels in fresh medium in all samples. However, for chip-C and chip-F, this reduction was much more prominent than for their respective disc substrates. From day 2, medium collected from both chip-C and chip-F samples reached phosphate levels similar to that of fresh medium (1.22 ​± ​0.25 ​mM). In addition, starting from day 2, the phosphate levels of the chip samples were statistically lower than the disc analogues. Generally, disc-F released a higher amount of phosphate than disc-C, which was statistically significant after day 1. Similarly, chip-F released more phosphate than chip-C during the first two days. However, no statistical differences were observed at any of the time points.

### 3.3 Release of Trolox from CDHA substrates

**Fig. 4:**
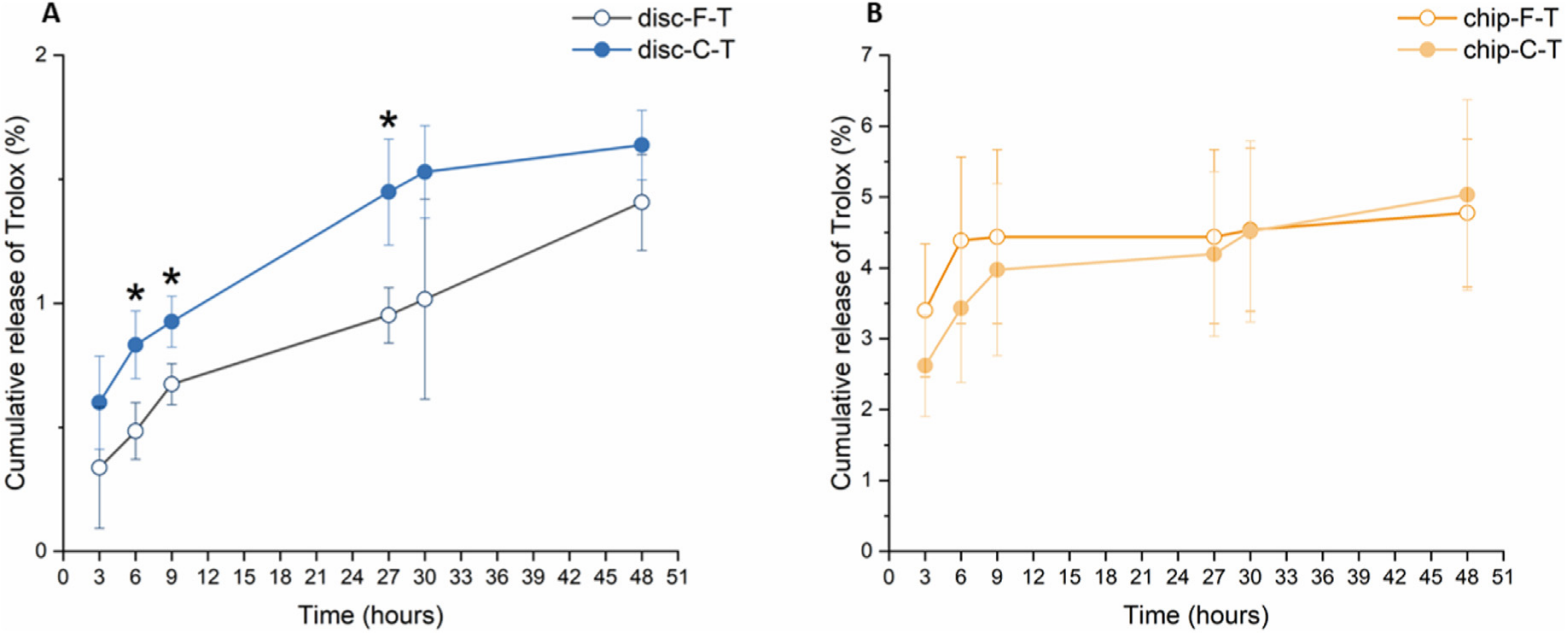
Cumulative release profile of Trolox from **(A)** disc-F and disc-C samples and **(B)** chip-F and chip-C samples. ∗ indicates p ​< ​0.05 between the samples.

For the disc samples, Trolox was released in a progressive manner, with the most rapid release being observed during the first 6 and 9 ​h for disc-C-T and disc-F-T samples, respectively (Fig. 4A). The slope of the release kinetics was higher within the first 9 ​h for both discs, coarse and fine. It was furthermore shown that disc-C-T released slightly more Trolox throughout the course of the experiment, which was shown to be statistically significant between 3 and 27 ​h (p-values indicated in Table S.M. 5). For the chip samples, the most rapid release of Trolox was also observed during the first 6 and 9 ​h for chip-C-T and chip-F-T, respectively (Fig. 4B). Subsequently, the release of Trolox slowed down and stabilized. Moreover, no significant differences between chip-F-T and chip-C-T were found during the experimental period. The overall release of Trolox from the discs during the period of 48 ​h was approximately 1.5%, while for chips, the total Trolox release reached 5% (1.4 ​± ​0.2, 1.6 ​± ​0.14, 4.8 ​± ​1.0 and 5.0 ​± ​1.3% for disc-F-T, disc-C-T, chip-F-T and chip-C-T, respectively).

### 3.4 Cell viability and cell proliferation

**Fig. 5:**
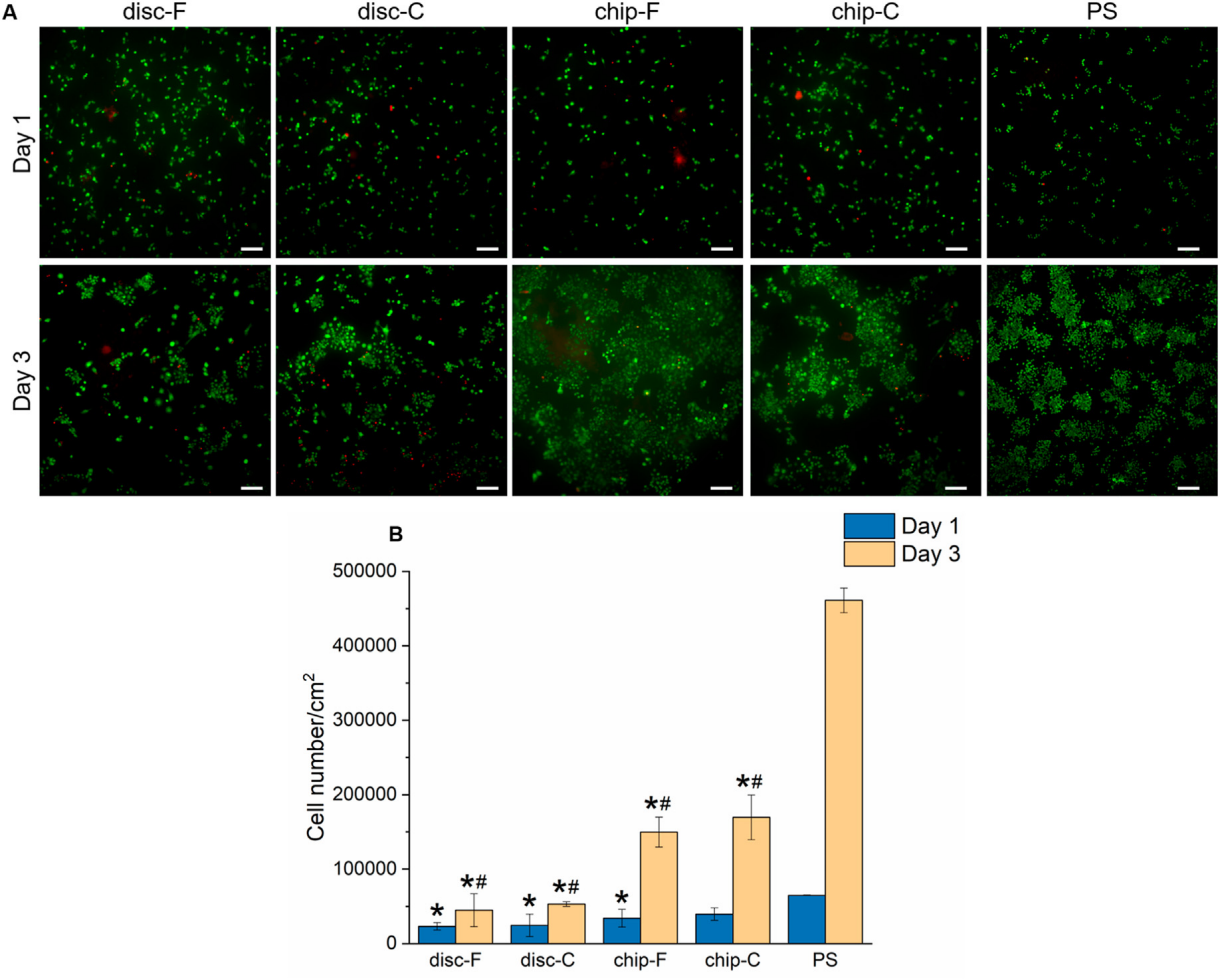
**(A)** RAW 264.7 ​cell viability determined by LIVE (green)/DEAD (red) staining after 1 and 3 days of culture on the disc-F, disc-C, chip-F, chip-C and PS samples. Scale bar corresponds to 100 ​μm. **(B)** Cell proliferation quantified with the LDH assay. The absorbance values were normalized to the surface area (*i.e.* 0.385 ​cm^2^ for the static samples and 0.975 ​cm^2^ on-chip)∗ indicates p ​< ​0.05 between the different CDHA substrates and PS control; # indicates p ​< ​0.05 between the chip and respective disc samples (*e.g.* difference between chip-F and disc-F); No statistically significant differences (p ​> ​0.05) were observed between F and C substrates of the same type (*e.g.* difference between chip-F and chip-C).

To assess the viability of RAW 264.7 ​cells on the disc and chip samples, LIVE/DEAD staining of the cells was performed. A vast majority of living cells was found on all substrates after 1 day of culture (Fig. 5A). As quantified by the LDH assay, no significant differences in cell number were observed among the CDHA substrates (Fig. 5B). However, on day 1, the cell number was higher for cells grown directly on the well plate surface, with significant differences observed for both disc samples and chip-F samples (disc-F, p ​= ​0.002; disc-C, p ​= ​0.003; chip-F, p ​= ​0.02). On day 3, the cells had proliferated on all substrates, with chip samples showing a significantly higher cell number than their disc analogues (F samples, p ​= ​0.003; C samples, p ​= ​0.006). As seen on day 1, the cell number was significantly higher for cells grown on the well plate surface (p ​< ​0.0005 for all conditions). In all conditions, the cells organized into clusters at the end of the culture period. Larger cell clusters were observed on the PS control.

### 3.5 TNF-α secretion

**Fig. 6:**
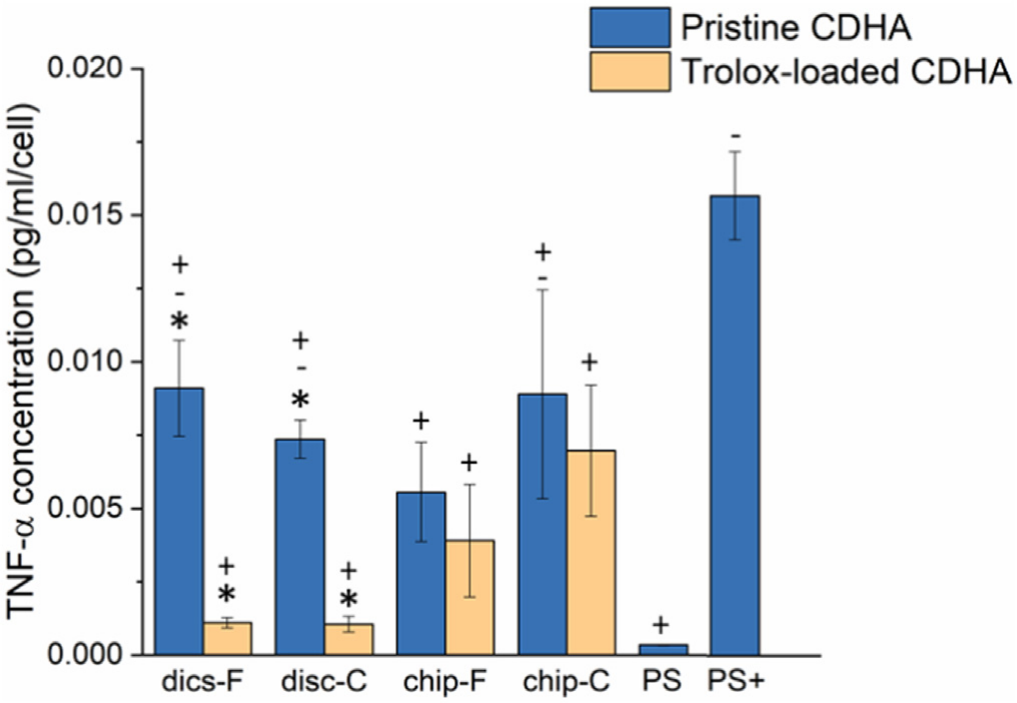
TNF-α secretion of RAW 264.7 ​cells grown on pristine (blue bars) and Trolox-loaded (yellow bars) CDHA substrates after 24 ​h of flow (or culture under static conditions). ∗ indicates p ​< ​0.05 between pristine and Trolox-loaded substrates; - indicates p ​< ​0.05 between CDHA substrates and PS; + indicates p ​< ​0.05 between CDHA substrates and PS+. No statistically significant differences (p ​> ​0.05) were observed between the chip and respective disc samples (*e.g.* difference between chip-F and disc-F) or between F and C substrates of the same type (*e.g.* difference between chip-F and chip-C).

To determine the inflammatory response of RAW 264.7 macrophages, TNF-α was quantified after 24 ​h of culture under static conditions and on-chip, both for pristine and Trolox-loaded CDHA. Looking at both the pristine and drug-loaded samples, no clear differences in TNF-α secretion were observed (Fig. 6), neither when comparing the topography (*i.e.* F versus C), nor when comparing static and dynamic conditions (*i.e.* discs versus chips). In general, a reduction in TNF-α was seen when the cells were grown on Trolox-loaded samples. These lower levels of TNF-α were most obvious when comparing pristine and Trolox-loaded discs, for which this difference appeared to be statistically significant (p ​= ​0.001 for both disc-F and disc-C; additional p-values can be found in Table S.M. 6).

### 3.6 Cell morphology

**Fig. 7:**
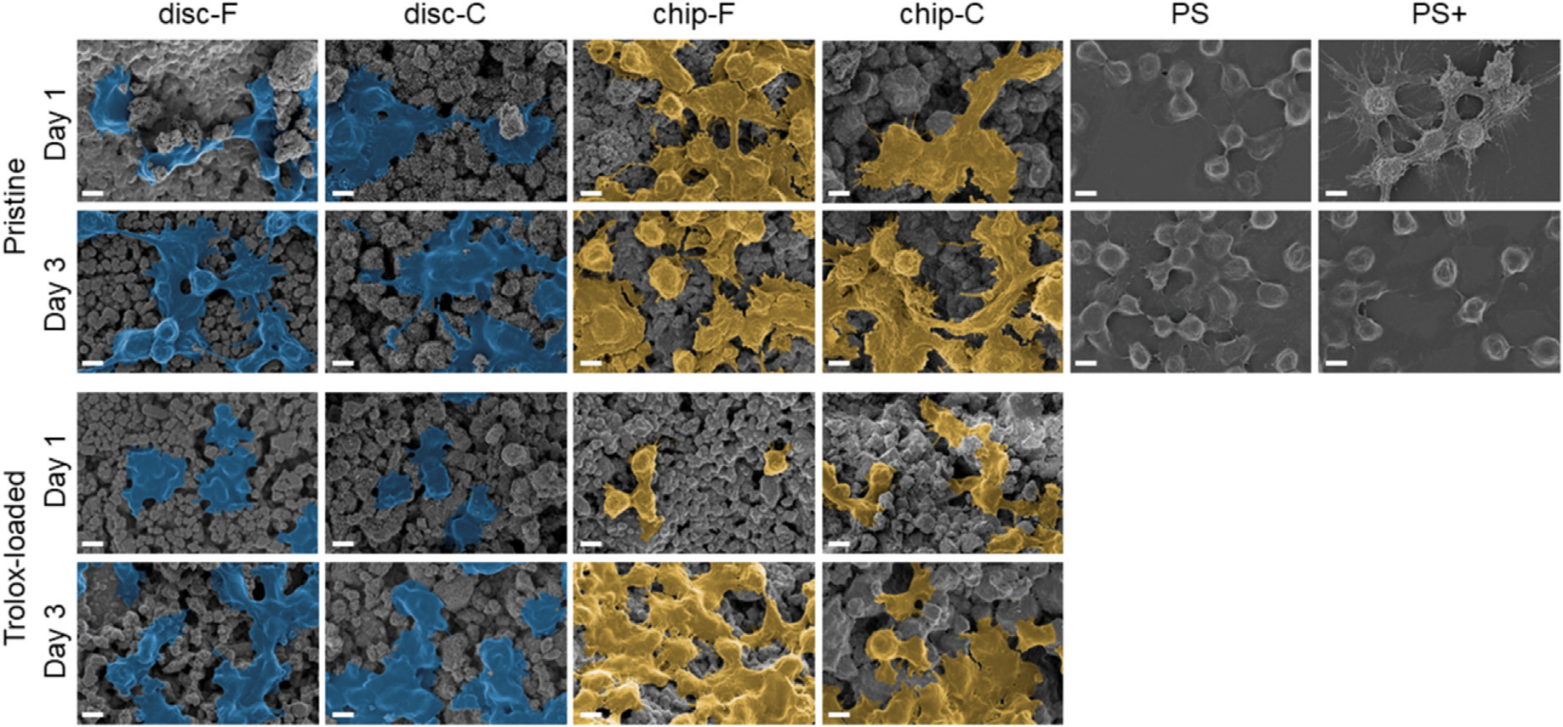
Morphology of RAW 264.7 ​cells on the different pristine and Trolox-loaded CDHA substrates after 1 and 3 days of culture. Scale bar corresponds to 5 ​μm.

On day 1, a clear difference in RAW 264.7 ​cell morphology was observed between the pristine disc samples (Fig. 7). Whereas cells on disc-F showed an elongated morphology, cells grown on disc-C appeared flat and spread. This difference between the disc samples became less pronounced on day 3. In general, the cells on the chip substrates were less flat than on their disc analogues and adopted both a round and spindle-like morphology. This variation in morphology between the chip and respective disc substrates could already be seen from day 1 for the C substrates and from day 3 for the F substrates. In addition, already from day 1, more cytoplasmic extensions were seen on the chip samples than for the discs. Although no drastic differences were observed between chip-F and chip-C samples, cells grown on chip-C were less round and more elongated than cells on chip-F. The cells on the Trolox-loaded samples were generally smaller and had fewer cytoplasmic extensions compared to the pristine samples. This was particularly clear for the Trolox-loaded disc samples, which exhibited the smoothest morphology. In addition, the cells on Trolox-loaded samples appeared as large fused clusters. Whereas this was already seen from 1 day on the discs, it became most apparent on day 3. For the chip samples, fusion was observed from day 3, although to a lesser extent than on the discs.

## 4 Discussion

The *in vitro* biological characterization of biomaterials is typically performed under static conditions. However, for biomaterials such as CDHA, such an environment can be problematic, as the highly reactive nature of these biomaterials can cause drastic alterations in the ionic composition of the cell culture medium, which is known to affect cell behavior [[18], [19], [20], [21], [22], [23]]. Furthermore, the type of CDHA microstructure, *i.e.* coarse or fine, has been shown to drive the ionic exchanges and protein sorption profiles [3,25], in addition to osteoinduction and resorbability [[15], [16], [17]]. Microfluidic technology has recently been proposed as an alternative tool to evaluate biomaterial-cell interactions [28]. As shown in our previous work, especially for bioactive materials such as CDHA, the option to provide (continuous) perfusion may highly influence the outcome of *in vitro* evaluations [27]. Taking this a step further, microfluidic technology could be particularly suitable for the characterization of drug-loaded biomaterials, as it enables the biological evaluation and characterization of drug release to take place under the same dynamic and potentially more physiologically relevant environment. However, in order to evaluate this, the results obtained from on-chip systems should be compared to the currently available methods, amongst which, testing biomaterials under static conditions in standard tissue culture plastic. Therefore, the aim of this work was to develop a microfluidic system that could be used to characterize both pristine and drug-loaded CDHA. We evaluated fluid-mediated interactions (*i.e.* ionic exchange and protein sorption) and studied the cell response of RAW 264.7 macrophage cells. In the second part of this study, we characterized the release of Trolox from the CDHA substrates and subsequently put this in biological context.

Protein sorption plays a key role in directing cell behavior and is known to be involved in processes such as cell adhesion, cell growth, cell differentiation and formation of the extracellular matrix [37]. Even though both F and C substrates have the same chemical composition, differences in protein sorption between these substrates were previously reported [3,20,25]. In agreement with Espanol et al. [3], we observed a larger albumin-FITC penetration region for the disc-C and chip-C samples compared to the F analogues (Fig. 2). As explained in their work, when considering protein sorption and penetration, both the available surface area and the presence of an interconnected pore network with relevant dimensions play an important role. Although both F and C substrates have a similar total porosity because the same liquid to powder ratio was used in both cases, there is a difference in pore size. Compared to F substrates, C substrates have a higher proportion of large voids. This is particularly relevant for protein penetration and can explain the larger penetration depth of albumin-FITC in the C samples. In addition, even though F substrates have a larger surface area, a large proportion of the pores are in the size range of the albumin molecule. This porosity range impedes albumin to pass through and indicates that not all of the surface is accessible for effective sorption of this protein. A larger level of protein sorption could be expected if a protein with smaller dimensions would be used. Apart from the differences between the F and C substrates, we also observed a larger penetration of albumin-FITC on-chip. This observation could be explained by the continuous medium renewal (and constant supply of fresh protein), which shortens the time needed to reach an equilibrium between CDHA and the medium. It could also be hypothesized that this effect is caused by the mechanical forces from the perfusion itself, which could facilitate protein penetration.

As mentioned before, for bioactive materials such as CDHA, ionic exchange is another highly relevant factor to consider when assessing its biological properties. As expected, when the CDHA substrates were maintained under static conditions (*i.e.* disc-F and disc-C), a clear uptake of calcium ions and release of phosphate ions was shown (Fig. 3) [20,22,23]. Initially, no significant differences between the disc and respective chip samples were observed. However, after 2–3 days, the chip samples approached or even reached the calcium and phosphate levels found in fresh medium. Generally, F substrates had a larger capacity to uptake calcium ions and release phosphate ions, regardless of whether being maintained under static or dynamic conditions. This trend was previously reported and could be attributed to the larger surface area of the F samples, compared to the C samples [20,38]. As for the protein sorption, the difference in ionic exchange between the disc and chip samples could be explained by the shorter time available to establish a chemical equilibrium on-chip. In addition, given the highly porous nature of CDHA, it is not unlikely that the bulk properties of the material have also contributed to our observations. As mentioned above, in this work we have correlated the static and dynamic conditions by matching the SA/V. However, the volume of one CDHA disc was approximately 1.5 times larger than the CDHA in one channel. This means that the volume of medium per volume of CDHA was approximately twice as large on-chip, which could explain why the calcium and phosphate concentrations in the medium obtained from chips reached values closer to that of fresh medium more quickly.

Calcium and phosphate are essential signaling molecules, known to regulate a large number of cell functions [39,40]. In the context of this work, it was for example shown that macrophages may detect changes in the extracellular calcium concentration via calcium-sensing receptors and that a change in calcium signaling is involved in stimulating cell proliferation [41]. It was also demonstrated that calcium-free medium limits the transcription of the c-fos gene, which is essential for cell proliferation and differentiation [42,43]. To the best of our knowledge, literature on the specific effects of phosphate on macrophages is limited. However, recent work has elucidated that elevated phosphate concentrations induce a phenotype resembling that of alternatively-activated M2 macrophages [44]. Whereas no drastic differences in cell number were observed between the cells grown on either of the CDHA substrates on day 1, cells grown on the chips proliferated to a much larger extent, emphasizing the effect that fluid-mediated interactions may have on cell behavior (Fig. 5). These findings are in line with a previous study, which aimed to separate the biological effects of the topographical features of CDHA and compositional changes in the cell culture medium [20]. As the authors reported, macrophage proliferation is particularly affected by fluid-mediated interactions, rather than by the topographical features. Interestingly, in their work, the difference between similar disc-F and disc-C substrates was also reflected in the cell studies, showing a decrease in RAW 264.7 ​cell number on disc-F samples compared to disc-C samples [20]. We did not observe this behavior, despite the similar trend in calcium and phosphate concentrations. It should however be noted that the SA/V in our work was almost four times lower and that different cell culture medium was used, which could potentially explain these differences. In fact, in other more comparable work, similar proliferation of RAW 264.7 ​cells on disc-F and disc-C substrates was shown, which is in line with our findings [38]. Generally, the cells cultured directly on the well plate surface showed superior proliferation at the two time points. CDHA has shown to reduce cell proliferation of a variety of cells *in vitro*, which has been attributed to a combination of alterations in the ionic environment and the microstructure of the substrates [[19], [20], [21],^45^]. Even though ionic imbalances can have a major impact on cell behavior, it should be mentioned that we have previously shown that the degree of cell proliferation on-chip does not fully correlate to differences in calcium and phosphate [27]. More specifically, whereas increased flow rates resulted in calcium and phosphate concentrations closer to that of fresh cell culture medium, MC3T3-E1 pre-osteoblast-like cells did not proliferate the most on the substrates that were exposed to the highest flow rate. This finding indicates that there are other factors in play, which together determine cell behavior on-chip. Apart from enhanced protein sorption and the ability to shield the cells from ionic imbalances, continuous medium renewal also implies a constant supply of nutrients and removal of waste to and from the cells [46]. Moreover, providing continuous flow results in exposure to shear stress, which, although at four orders of magnitude higher, has shown to affect macrophage activation [47].

To further investigate cell behavior, we evaluated the secretion of TNF-α from RAW 264.7 ​cells culture on the different substrates. For the pristine samples, no clear differences in TNF-α secretion were observed, neither between the discs and the chips, nor between F and C substrates of the same type (Fig. 6). Interestingly, in previous work, TNF-α secretion was shown to be higher on similar disc-C substrates when compared to disc-F substrates [38]. One possible explanation for this discrepancy is the difference in basic experimental set-up, such as differences in SA/V ratio. Moreover, whereas higher TNF-α secretion was reported, the authors concluded an overall pro-inflammatory status of RAW 264.7 ​cells on both F and C substrates, based on the evaluation of other pro-inflammatory cytokines. We acknowledge that the assessment of only TNF-α is a limitation of our study and that a more in-depth characterization of complementary cytokines, and cell membrane markers expressed by the cells over the culture period would be necessary to make stronger statements regarding the cells’ phenotype. In addition to cytokine secretion, macrophage morphology can be linked to its polarization state. A flat and spread out pancake-like shape is generally associated with a pro-inflammatory (M1) phenotype and a more elongated spindle-like shape with an anti-inflammatory (M2) phenotype [48]. Among our pristine substrates, cells grown on disc-C showed a markedly different morphology from the cells grown on the other samples (Fig. 7). The cells on disc-C were predominantly flat and spread out, matching the classical morphology of M1-polarized cells. Previous work has shown an increase in the formation of reactive oxygen species (ROS) for RAW 264.7 ​cells cultured on similar disc-C substrates, suggesting a more inflammatory environment than on disc-F [20]. When the cells were subjected to flow (*i.e.* chip-F and chip-C), both elongated and round cells were seen. This heterogeneity has been more often observed for M2-induced macrophages and indicates a more anti-inflammatory environment for RAW 264.7 ​cells on-chip [[49], [50], [51]].

In the second part of this study, Trolox was added to the CDHA substrates and its release and biological effects were evaluated. In the context of their function as a drug carrier, CPCs can be classified as non-swellable monolithic materials [4]. It can be assumed that drug release is mainly controlled by diffusion, which implies that the microstructure of the carrier has a crucial role in drug liberation. As described above, F and C substrates differ in terms of pore size and pore size distribution. In line with this, we have shown a slightly higher release of Trolox for disc-C-T samples compared to disc-F-T samples (Fig. 4). The overall release profile was similar for disc-F-T and disc-C-T, showing progressive release throughout the course of the experiment and the most rapid release during the first 6–9 ​h. Overall, this general trend correlates well to the release of Trolox from brushite cements, for which a similar release profile was seen, although with higher levels of Trolox being released [52]. Given the higher solubility of brushite cements, this is however not surprising [53]. Furthermore, our findings are in agreement with a previous study on similar CDHA substrates, in which the release of doxycycline was highly dependent on the pore size and total porosity [54]. In this work, CDHA coarse substrates released less than 4% doxycycline during the first 50 ​h and less than 10% for a total of 100 ​h, which is well in line with our results. Our discs released approximately 1.5%, while chips Trolox cumulative release reached 5%. The larger surface area of disc-F could explain the absence of significant differences at the earliest time point (*i.e.* 3 ​h). More specifically, as a larger surface area of the disc-F samples is exposed to medium, more Trolox could be released, compensating for the differences in pore size between F and C. On-chip, Trolox was released during the first 6–9 ​h, after which a plateau was reached. Interestingly, no obvious differences were observed between chip-F-T and chip-C-T. Whereas the reason remains elusive, we hypothesize that this could be due to a perfusion-associated effect, which may influence the effect of differences in pore size and surface area. In other words, as the medium is continuously renewed and no chemical equilibrium is reached, it is likely to assume that the effect of the larger surface area of F substrates may contribute to a larger extent. It would therefore be plausible that the difference in surface area is balancing the difference in pore size, resulting in similar release profiles.

From the drug release data, we can conclude that by the time the cells are seeded on the samples (*i.e.* after overnight incubation with medium), the drug release on-chip has already stabilized (Fig. 4B). It should be emphasized that the data depicted in Fig. 4B represents the cumulative release of Trolox. However, on-chip, the medium is continuously washed out, meaning that the cells are not exposed to increasing concentrations of Trolox, but rather to decreasing amounts of Trolox over time. It is therefore not surprising that no significant differences in TNF-α secretion were observed between cells maintained on pristine or drug-loaded chips (Fig. 6). When comparing the discs, a significant reduction in TNF-α was found for the cells cultured on Trolox-loaded samples. This finding suggests a relation between Trolox and the secretion of TNF-α, which could be explained by Trolox's antioxidant activity and the interaction between ROS and TNF-α [55]. More specifically, while cytokines can induce the generation of ROS, it has also been shown that ROS are able to stimulate the production of pro-inflammatory cytokines by activating nuclear factor κB (NF-κB), which is a transcription factor with key roles in a variety of processes, including inflammation and immunity [56]. Overall, our finding correlates well with previous work, in which it was shown that Trolox could not only reduce reactive oxygen species (ROS), but also lower the levels of pro-inflammatory cytokines, including TNF-α [57,58].

When looking at the morphology, the cells on the Trolox-loaded samples were generally smaller, with fewer cytoplasmic extensions than the cells on the pristine analogues (Fig. 7). This reduction in cytoplasmic extensions was most obvious for the Trolox-loaded discs, on which the cells looked smoother, already from day 1. A small and round morphology lacking cytoplasmic extensions was previously reported for unstimulated macrophages [59]. In addition to the round morphology observed on day 1, after 3 days on Trolox-loaded substrates, the cells appeared fused. Whereas this could be a sign of foreign body giant cell (FBGC) formation [60], it should be emphasized that our data alone is not sufficient to confirm this with certainty [5]. Interestingly, α-Tocopherol, which is one of the isoforms of vitamin E, is able to convert human macrophages to fusion-competent macrophages [61,62]. Given that Trolox is a synthetic analogue of vitamin E, it could be hypothesized that Trolox would also be able to induce fusion of the cells. As also mentioned above, in order to make more conclusive statements regarding FBGC formation, additional markers should be evaluated. Noteworthy, whereas macrophage fusion was already obvious from day 1 on the Trolox-loaded discs, we did not observe this effect until day 3 for the respective chips. These findings are in line with the drug-release data, which showed higher exposure to Trolox for the cells maintained on the discs. Moreover, it seems plausible that a continuous exposure to lower levels of Trolox could, although with a delay, still induce macrophage fusion over time. At first glance, the fusion of cells (and potential formation of FBGCs), which are historically considered a hallmark of chronic inflammation, might seem in contrast with Trolox's known antioxidant properties and our results (*i.e.* reduction in TNF-α). However, the same authors who showed the potency of α-Tocopherol-induced macrophage fusion have also demonstrated that the incorporation of vitamin E in poly(etherurethane) biomaterials significantly reduced its degradation [63]. This finding was explained by the more contemporary view on FBGCs, which argues that these cells can adopt both M1 and M2 phenotypes and could therefore contribute to healing and tissue remodeling [62,64]. Lastly, it should be noted that it was also shown that Trolox, unlike α-Tocopherol, was not able to induce fusion of human macrophages [62]. Even though our findings are not completely in line with that observation, this discrepancy could potentially be explained by the difference in cell source and interspecies differences, which have previously been shown for human and murine macrophages. It is for example known that macrophages of murine and human origin can respond differently to the same cytokines, can have different M1 and M2 phenotype-specific markers and show differences in the expression of receptors and ligand specificity [65].

## 5 Conclusion

In this work, a microfluidic platform was developed to investigate the biological properties of nanostructured CDHA, both in pristine form and when drug-loaded. We demonstrated that a continuous supply of medium enhanced protein sorption and compensated for drastic ionic fluctuations. This was reflected in the cell studies, which showed a high degree of cell viability and enhanced cell proliferation for the cells maintained on-chip. Whereas no differences in TNF-α secretion were found, assessment of macrophage morphology suggested a more anti-inflammatory environment on-chip. We successfully characterized the release of Trolox on-chip and subsequently examined the influence of Trolox on the secretion of TNF-α and its effect on the morphology of RAW 264.7 ​cells. Overall, this study highlights the promise of microfluidics for the characterization for biomaterials, especially for those that are highly reactive. In addition, we have shown that this approach could be a promising route towards more standardized and potentially more physiologically relevant characterization of drug-loaded biomaterials, as it allows for both the evaluation of the biological properties of biomaterials and drug release to be conducted under the same *in vitro* test setting.

## Credit author statement

S-S.D.C., A.D-E., G.M.: Conceptualization; S-S.D.C., A.D-E., M.G., G.M.: Data curation; S-S.D.C., A.D-E.: Formal analysis; G.M., M.T.: Funding acquisition; S-S.D.C., A-R.A., A.D-E., M.G., G.M.: Investigation; S-S.D.C., A-R.A., A.D.-E., M.G., M-P.G., G.M.: Methodology; S-S.D.C., A.D-E., G.M.: Project administration; M-P.G., M.T., G.M.: Resources; A.D-E, M.T., G.M.: Supervision; S-S.D.C., A-R.A., A.D-E., M.G., G.M.: Validation; S-S.D.C., A.D-E.: Visualization; S-S.D.C: Writing - original draft; S-S.D.C., A-R.A., A.D-E., M.G., M-P.G., M.T., G.M.: Writing - review & editing.

## Data availability

The raw/processed data required to reproduce these findings cannot be shared at this time due to technical or time limitations.

## Declaration of competing interest

The authors declare that they have no known competing financial interests or personal relationships that could have appeared to influence the work reported in this paper.
