# Supplementary material for: A microfluidic-based approach to investigate the inflammatory response of macrophages to pristine and drug-loaded nanostructured hydroxyapatite
Source: Mater Today Bio. 2022 Jul 7;16:100351. doi: 10.1016/j.mtbio.2022.100351 (PMC9294551; doi:10.1016/j.mtbio.2022.100351)
Supplement: Multimedia component 1 [file mmc1.docx]

Supplementary Material

**A microfluidic-based approach to investigate the inflammatory response of macrophages to pristine and drug-loaded nanostructured hydroxyapatite**

Sarah-Sophia D. Carter^a^, Abdul-Raouf Atif^a^, Anna Diez-Escudero^b^, Maja Grape^a^, Maria-Pau Ginebra^c,d,e^, Maria Tenje^a^ and Gemma Mestres^a,*^

^a^Division of Biomedical Engineering, Department of Materials Science and Engineering, Science for Life Laboratory, Uppsala University, 751 22, Uppsala, Sweden

^b^Ortholab, Department of Surgical Sciences—Orthopaedics, Uppsala University, 751 85 Uppsala, Sweden

^c^Biomaterials, Biomechanics and Tissue Engineering Group, Departament de Ciència i Enginyeria de Materials, Universitat Politècnica de Catalunya (UPC), 08930 Barcelona, Spain

^d^Barcelona Research Center in Multiscale Science and Engineering, Universitat Politècnica de Catalunya, 08930 Barcelona, Spain

^e^Institute for Bioengineering of Catalonia (IBEC), Barcelona Institute of Science and Technology (BIST), Baldiri Reixac 10-12, 08028 Barcelona, Spain

*Corresponding author: Gemma Mestres

Postal Address: Department of Materials Science and Engineering

Uppsala University, Box 534, 751 21 Uppsala, Sweden

E-mail: [gemma.mestres@angstrom.uu.se](mailto:gemma.mestres@angstrom.uu.se)

**1. Particle size distribution of the Fine and Coarse cement powder**

The particle size of the two starting alpha-tricalcium phosphates was analyzed through laser diffraction (Mastersizer 3000, Malvern Instruments). The powders, Fine and Coarse, were dispersed in absolute ethanol until an obscuration of the laser of approximately 10% was achieved, and analyzed using Mie correction model, with a refractive index of 1.360 for ethanol and 1.630 for the α-TCP.

**Table S.M. 1.** Volume percentage values from the particle size distribution of the Fine and Coarse cement powder, from which the calcium-deficient hydroxyapatite (CDHA) substrates were prepared.

|  | **Particle size (µm)**  **Fine Coarse** | |
| --- | --- | --- |
| d_10_ | 0.90 | 1.45 |
| d_50_  d_90_ | 2.89  12.5 | 7.36  22.2 |

**2. The effect of Trolox-loading on the microstructure of CDHA**

To examine the effect of the incorporation of Trolox on the characteristic microstructure of CDHA, SEM was performed. Pristine and Trolox-loaded disc substrates (*i.e.* disc-F, disc-C, disc-F-T and disc-C-T) were prepared as described in sections 2.1.1. and 2.1.3 and gold-coated (2-4 nm) (Emitech SC7640, Quorum technologies). At least three random sites were imaged on one sample of each type. As can be seen in Figure S.M.1., no obvious differences were observed between pristine and Trolox-loaded samples, which suggests that Trolox does not affect the microstructure of our samples.


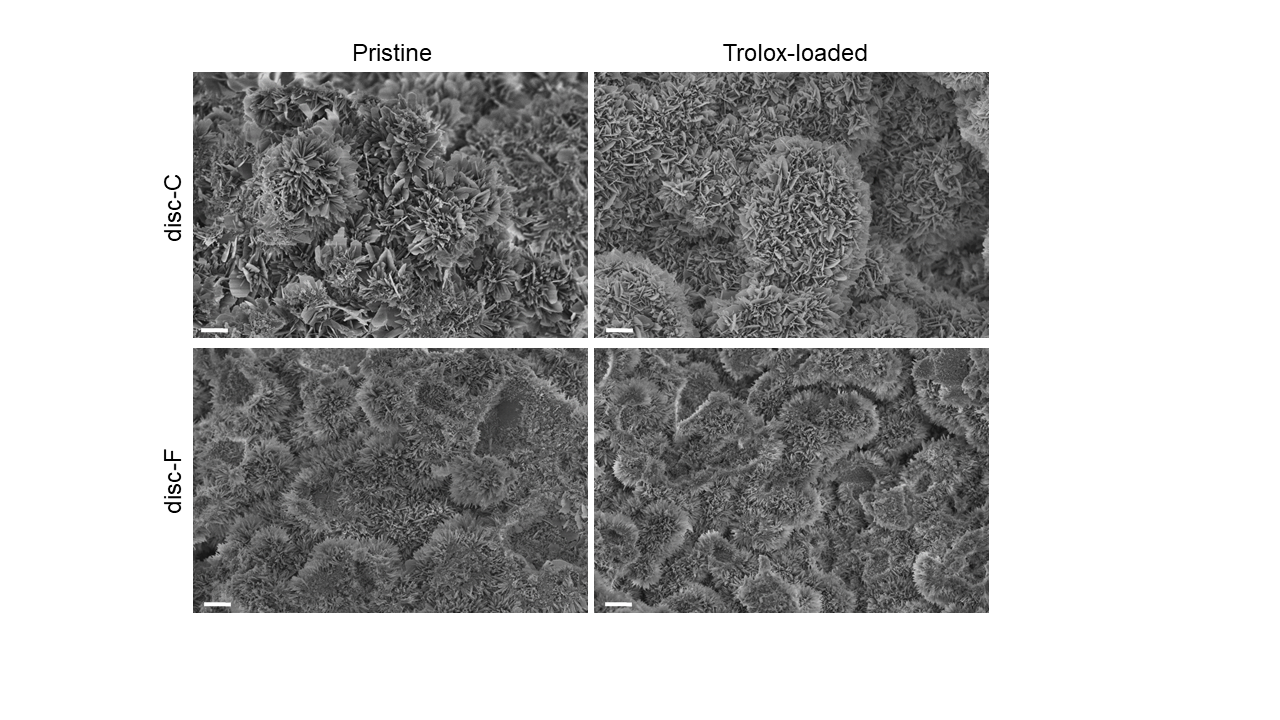


**Figure S.M. 1.** SEM images of pristine (*i.e.* disc-F and disc-C) and Trolox-loaded (*i.e.* disc-F-T and disc-C-T) samples. Scale bar corresponds to 1 µm.

**3. The effect of sample storage on the degradation of Trolox**

To evaluate whether sample storage affected the Trolox concentration, a standard curve with known concentrations of Trolox (0-500 µM) was prepared, after which the absorbance was measured in a microplate reader at 290 nm. The samples were either measured directly (fresh solution) or after storage for 48 hours at 2-8 °C (TECAN, Spark®). As can be seen in Figure S.M. 2, no differences were observed for any of the concentrations, indicating that samples storage for 48 hours at 2-8 °C does not affect the concentration of Trolox.





**Figure. S.M. 2.** Absorbance values of known Trolox concentrations when measured directly after preparing the solutions (fresh solutions) and after storage for 48 hours at 2-8 °C.

**4. RAW 264.7 cells attachment**

Prior to evaluating the performance of the different systems (disc *vs.* chip), murine RAW 264.7 cells were evaluated for their attachment. Briefly, 20,000 cells/cm^2^ were seeded both on the discs (ø = 7 mm) placed in 48-well plates and on the chips. As a control, the cells were seeded directly on the polystyrene surface of the well plate (PS). After a static incubation of 2 hours, the samples were washed (x2) with transparent Minimum Essential Medium (MEM) (Gibco™, ref. nr. 51200046). The samples were subsequently stained with LIVE/DEAD staining (calcein-AM/propidium iodide (PI); Invitrogen^TM^, ref. nr. C3099 and ref. nr. P3566, respectively) at a final concentration of 1 µg/mL (in transparent MEM) and incubated for 15 minutes at standard cell culture conditions while protected from light. Afterwards, the samples were washed with transparent MEM (x3) and imaged using a fluorescence microscope (Olympus, IX73; excitation/emission = 494/518 nm and 595/615 nm for calcein and PI, respectively). Living cells were visualized in green and dead cells in red.


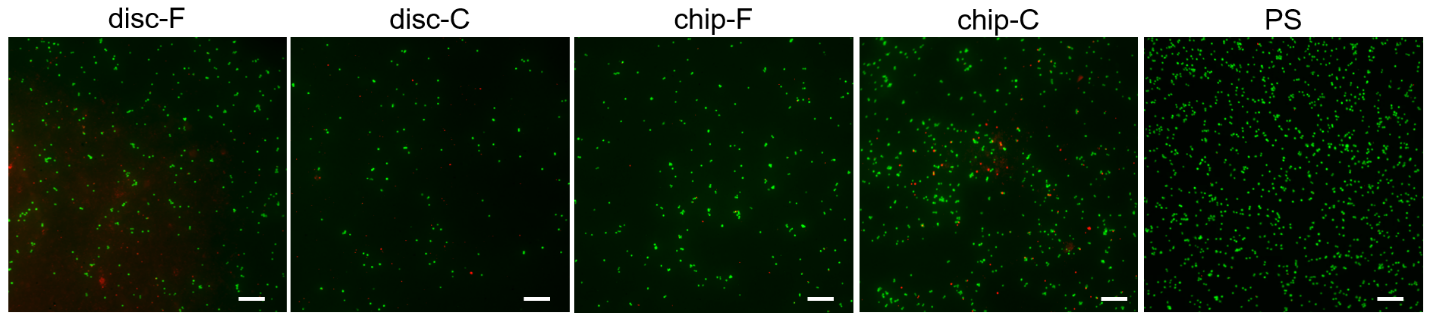


**Figure. S.M. 3.** RAW 264.7 cells attachment after 2 hours of incubation determined by LIVE (green)/DEAD (red) staining. Scale bars correspond to 100 µm.

**5. Summary of the statistical analysis**

**Table S.M. 2.** p-values corresponding to the data plotted in Figure 2B, in which the penetration depth of albumin-FITC was evaluated (significance level of α = 0.05).

|  | **1 hour** | **6 hours** | **24 hours** |
| --- | --- | --- | --- |
| disc-F – disc-C | p > 0.05 | P = 0.005 | P = 0.001 |
| disc-F – chip-F | p = 0.004 | p > 0.05 | P = 0.005 |
| disc-C – chip-C  chip-F – chip-C | P = 0.002  p = 0.007 | P = 0.002  P = 0.002 | p < 0.0005  p < 0.0005 |

**Table S.M. 3.** p-values corresponding to the data plotted in Figure 3A, in which the concentration of calcium in the cell culture was evaluated (significance level of α = 0.05).

|  | **Day 1** | **Day 2** | **Day 3** | **Day 4** |
| --- | --- | --- | --- | --- |
| disc-F – fresh medium | p < 0.0005 | p < 0.0005 | p < 0.0005 | p < 0.0005 |
| disc-C – fresh medium | p < 0.0005 | p < 0.0005 | p < 0.0005 | p = 0.02 |
| chip-F – fresh medium | p < 0.0005 | p < 0.0005 | p < 0.0005 | p < 0.0005 |
| chip-C – fresh medium | p < 0.0005 | p > 0.05 | p > 0.05 | p > 0.05 |
| disc-F – disc-C | p > 0.05 | p = 0.02 | p = 0.03 | p > 0.05 |
| disc-F – chip-F | p > 0.05 | p > 0.05 | P = 0.001 | p < 0.0005 |
| disc-C – chip-C  chip-F –chip-C | P > 0.05  p > 0.05 | p = 0.002  p < 0.0005 | p < 0.0005  p < 0.0005 | p < 0.0005  P = 0.03 |

**Table S.M. 4.** p-values corresponding to the data plotted in Figure 3B, in which the concentration of phosphate in the cell culture was evaluated (significance level of α = 0.05).

|  | **Day 1** | **Day 2** | **Day 3** | **Day 4** |
| --- | --- | --- | --- | --- |
| disc-F – fresh medium | p < 0.0005 | p < 0.0005 | p < 0.0005 | p = 0.002 |
| disc-C – fresh medium | p = 0.002 | p = 0.009 | p = 0.003 | p > 0.05 |
| chip-F – fresh medium | p < 0.0005 | p > 0.05 | p > 0.05 | p > 0.05 |
| chip-C – fresh medium | p = 0.003 | p > 0.05 | p > 0.05 | p > 0.05 |
| disc-F – disc-C | p > 0.05 | p = 0.047 | p = 0.008 | p = 0.045 |
| disc-F – chip-F | p > 0.05 | p = 0.001 | p < 0.0005 | p < 0.0005 |
| disc-C – chip-C  chip-F –chip-C | p > 0.05  p > 0.05 | p = 0.006  p > 0.05 | p = 0.001  p > 0.05 | p = 0.003  p > 0.05 |

**Table S.M. 5.** p-values corresponding to the data plotted in Figure 4A, in which the release of Trolox from the disc substrates was evaluated (significance level of α = 0.05). Noteworthy, we only presented the samples and time points for which p < 0.05 was found.

|  | **6 hours** | **9 hours** | **27 hours** |
| --- | --- | --- | --- |
| disc-F-T – disc-C-T | p = 0.005 | p = 0.009 | p = 0.01 |

**Table S.M. 6.** p-values corresponding to the data plotted in Figure 6, in which the secretion of TNF-α was evaluated on the **(A)** pristine and **(B)** Trolox-loaded samples (significance level of α = 0.05). In **(C)** we compared the pristine and Trolox-loaded samples.

| **A. Pristine samples** | |
| --- | --- |
| disc-F – disc-C | p > 0.05 |
| disc-F – chip-F | p > 0.05 |
| disc-C – chip-C  chip-F – chip-C  disc-F – PS  disc-C – PS  chip-F – PS  chip-C– PS  disc-F – PS+  disc-C – PS+  chip-F – PS+  chip-C– PS+ | p > 0.05  p > 0.05  p = 0.002  p = 0.021  p > 0.05  p = 0.002  p = 0.015  p = 0.007  p = 0.001  p = 0.013 |

| **B. Trolox-loaded samples** | |
| --- | --- |
| disc-F-T – disc-C-T | p > 0.05 |
| disc-F-T – chip-F-T | p > 0.05 |
| disc-C-T – chip-C-T  chip-F-T – chip-C-T  disc-F-T – PS  disc-C-T – PS  chip-F-T – PS  chip-C-T – PS  disc-F-T – PS+  disc-C-T – PS+  chip-F-T – PS+  chip-C-T – PS+ | p > 0.05  p > 0.05  p > 0.05  p > 0.05  p > 0.05  p > 0.05  p < 0.0005  p < 0.0005  p = 0.001  p = 0.001 |

| **C. Pristine – Trolox-loaded** | |
| --- | --- |
| disc-F – disc-F-T | p = 0.001 |
| disc-C – disc-C-T | p = 0.001 |
| chip-F – chip-F-T  chip-C – chip-C-T | p > 0.05  p > 0.05 |
